# A scope review on the global impact of COVID-19 lockdown on adolescents' health

**DOI:** 10.4314/ahs.v21i4.4

**Published:** 2021-12

**Authors:** Olayinka Ilesanmi, Aanuoluwapo Afolabi, Ayi Kwaghe

**Affiliations:** 1 University of Ibadan, College of Medicine, Department of Community Medicine; 2 University College Hospital Ibadan, Department of Community Medicine; 3 Federal Ministry of Agriculture and Rural development, Department of Veterinary and Pest Control Services; 4 Nigerian Field Epidemiology and Laboratory Training Programme; National Tuberculosis, Leprosy, and Buruli Ulcer Control Programme

**Keywords:** Adolescent health, Coronavirus, COVID-19 lockdown

## Abstract

**Background:**

The implementation of COVID-19 lockdown measures across the globe could affect adolescents' health.

**Objective:**

This review was conducted to assess the impact of the COVID-19 lockdown on the health of the adolescents.

**Methodology:**

We conducted this study using the scope reviews methodological framework. We searched for articles on the effects of COVID-19 lockdown among adolescents on four databases; MedLine, PubMed, Directory of Open Access Journals and Google Scholar. Screening of articles was done for relevance to the study objective.

**Results:**

The positive effects of the COVID-19 lockdown on adolescents included increased physical activity for adolescents 17 years and below, increased resource mobilization for healthy lifestyle, and psychosocial support programs for schools. The negative effects of the lockdown period were decreased physical activity with resulting disruptive sleep patterns; increased screen time, behavioral addictive disorder from excessive use of the internet, increased levels of domestic abuse, and worsening of existing mental health disorders. Overall, the COVID-19 lockdown period has had considerable adverse effects on adolescents' health.

**Conclusion:**

To curb the negative effects of the COVID-19 lockdown, we recommend parental supervision of adolescents' screen time. Adolescent health should be prioritized by policymakers to ensure that future lockdown does not adversely affect them.

## Introduction

The novel Coronavirus disease (COVID-19) has infected and caused many deaths globally^1^. COVID-19 was declared a public health emergency of international concern in March, 2020 by the World Health Organization (WHO)^2^,^3^. The high rate of infectiousness and associated case fatality subsequently led to the implementation of lockdown measures by many countries around the globe^4^. Preliminary data in countries with lockdown measures indicating a 50% decline in the number of COVID-19 cases have reported relative protection of adolescents^2^,^5^. Implemented lockdown measures resulted in the closure of schools, cancellation of out-of-home activities, and a denial of physical interaction of adolescents with their peers and tutors^3^. Due to these changes, adolescents have had to share restricted home settings and limited resources with their parents and siblings all day long^5^. To accommodate the new developments, adolescents developed coping strategies and new routines during the COVID-19 lockdown period for optimal performance^5^,^6^.

Health is the overall state of physical, mental, social, and emotional wellbeing, and not a mere absence of illnesses or infirmities^7^. Literature on the effects of the COVID-19 lockdown have reported mental, physical, and emotional health implications among the young and old^8^–^10^. The impracticability of some of the hobbies of these adolescents as a result of the lockdown made them vulnerable to binge on playing video games which could ultimately translate to addictive behavior^10^. Such behavioral addiction could result to attention deficit following the return to normalcy as reported by other studies^10^,^11^. In relation to the internet gaming disorder in the Diagnostic and Statistical Manual of mental sciences-V (DSM-5) and International classification of Diseases-11 (ICD-11), physical inactivity predisposes to frequent binge eating and obesity^10^,^12^. These are likely implications of the COVID-19 lockdown. These could have profound effects on adolescents' health and wellbeing in years to come. Hence, an assessment of the effect of the COVID-19 lockdown on adolescent health paramount for planning the management of the effects of future pandemics.

The WHO recommends rapid reviews in the development of guidelines for public guidance in dealing with COVID-19^13^. This is essential to provide up-to-date information regarding the infection, its effects, and coping strategies. The negative impact of the lockdown on adolescents' health and wellbeing cannot be overlooked^3^. The vulnerability of adolescents to exacerbated effects of such times as this necessitate the need for the assessment of the COVID-19 lockdown on their health. Therefore, we undertook a scope review on the impact of the COVID-19 lockdown on adolescents' health.

## Methods

### Study Design

We conducted this study using the scope reviews methodological framework developed by Arksey and O'Malley and modified by several studies^14^–^16^. The framework involves six steps in the analysis which are: a clearly defined research question; identification of relevant studies using electronic databases, reference lists, hand searches, and gray literature; study of the selection process, charting the process of data extraction, collating, summarizing, and reporting the results; and an optional consultation exercise^17^. The scope review identifies available evidence in the clarification of key concepts, examines study design on a subject matter, identifies associated key elements, identifies and analyzes knowledge gaps, and sets the stage for performing a systematic review^14^. In the context of the COVID-19 pandemic, a scope review could help to identify issues relating to the effects of the COVID-19 outbreak across different groups in the population.

### Search Strategy

A literature search of available evidence was undertaken on the effects of the COVID-19 lockdown among adolescents on four databases; MedLine, PubMed, Directory of Open Access Journals (DOAJ), and Google Scholar. The selection of these databases was based on their indexing of a wide range of journals. Keywords were used in the search strategy with the use of Boolean operator ‘AND’: We selected studies that focused on the effects of the COVID-19 lockdown among adolescents or the effects of social isolation due to COVID-19 on adolescents for this review. We excluded non-COVID-19 articles and COVID-19 articles that were not related to the study. Keywords used for the search included: “COVID-19 AND Adolescent”; “Coronavirus AND Adolescent”; “COVID-19 Lockdown AND Adolescent”; “COVID-19 Isolation AND Adolescent”; “COVID-19 AND Youngsters”; and “COVID-19 In-home stay AND Adolescent”. A threestep strategy was adopted in the literature search:

**Step One:** A search of Medline, PubMed, and DOAJ and Google Scholar databases was used to identify index terms and text words contained in the title and abstract.

**Step Two:** Identified keywords and index terms were used to prompt search on included databases.

**Step Three:** Additional literatures were obtained through a search of the reference lists from relevant articles.

**Figure 1:**
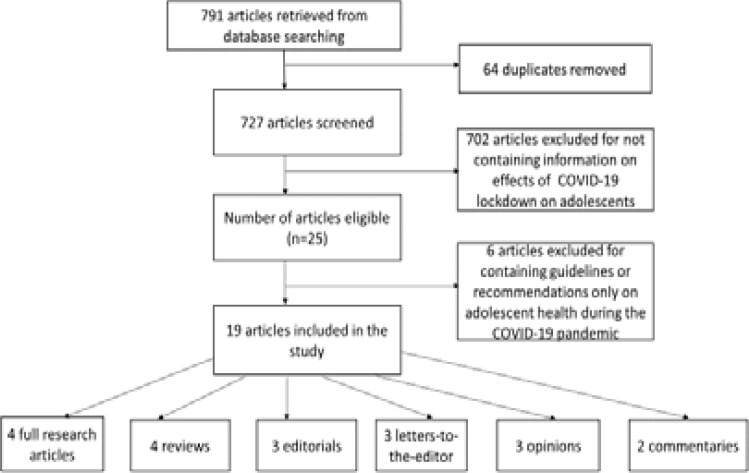
Flow chart for the scope review process

A total of 791 articles were retrieved through database searching, followed by the removal of 64 duplicates. A total of 727 articles remaining were screened, out of which 702 articles were excluded for not containing information on the effects of COVID-19 lockdown on adolescents. A total of 25 articles were eligible for the study. Six articles were excluded for containing only guidelines or recommendations on adolescent health during the COVID-19 pandemic. In all, 19 articles were included in the study; 4 full research articles, 4 reviews, 3 editorials, 3 letters to the editor, 3 opinion articles, and 2 commentaries (Figure 1).

### Ethical Considerations

Not applicable

## Results

The results obtained in this review are as shown in Table 1. We identified the positive and negative effects of the COVID-19 lockdown on adolescents' health. Seven studies identified the positive effects of the COVID-19 lockdown on adolescents' health. A decline in fast food intake, and an increased intake of legumes, fruits, and vegetables was observed. Increased physical activity, reduced sedentary behavior, and increased effectiveness of online schooling in improving mental and physical health were also identified as the positive effects of the COVID-19 lockdown on adolescents.

**Table 1:** Summary of the positive and negative effects of the COVID-19 lockdown on adolescents

S/N	Author	Source Location	Aims	Study Design/Type	Outcome of Interest	Negative effects of COVID-19 lockdown	Positive effects of COVID-19 lockdown
A.	Guessom et al., 2020^17^	Not Stated (NS)	Assessment of the impact of the COVID-19 pandemic and lockdown on adolescent psychiatric disorders	Narrative review	The risk of worsening psychiatric disorders in the COVID-19 pandemic	- Increased risk of psychological trauma, anxiety, and depression -Decreased physical activity, irregular sleep patterns, more screen time, and less appropriate diets	-
B.	Ruiz- Roso et al., 2020^1^	Italy, Spain, Chile, Colombia and Brazil	Assessment of the effects of COVID-19-induced confinement policies on self- reported nutritional habit modifications compared with their usual diet and dietary guidelines.	Full research/ Cross-sectional	Nutritional consequences of COVID-19 lockdown among adolescents	- Increase in the average intake of fried and sweet foods	-Reduction of fast food intake -Increased intake of legumes, fruits, and vegetables
C.	Magaritis et al., 2020^3^	NS	Determination of whether why, or how to deal with short- or medium-term lockdown-related Physical inactivity (PI) and Sedentary Behavior (SB) increase in young children and adolescents based on Anses' benchmark.	Commentary	Lockdown results in a decrease in Physical Activity (PA) in young people	-Risk of cardiometabolic complications and high levels of anxiety associated with PI and SB	-Increased PA and reduced SB for children and adolescents aged 6 to 17 years
D.	Nagata et al., 2020^18^	NS	Implications of excessive screen time during the COVID-19 pandemic	Opinion	Consequences of excessive screen time during the COVID-19 lockdown among adolescents	-Excessive screen time is associated with poor sleep -Increased screen time may also further exacerbate risk for depression, anxiety, suicide, and inattention among children and adolescents	
E.	Tornese et al., 2020^19^	NS	Evaluation of the changes in glycemic control and the role of PA at home using a hybrid closed loop (HCL) system	Full research article/ Retrospective cohort	Changes in glycemic control during the COVID-19 lockdown		-Good glycemic control at baseline did not worsen at two weeks of lockdown -Improvement in Time in Range (TIR) and Time Below Range (TBR) after two weeks of lockdown
F.	Ko and Yen, 2020^9^	NS	Assessment of the impact of COVID-19 on gaming disorder: monitoring and prevention	Letter-to-the- Editor	Monitoring and prevention of gaming disorder (GD) during the COVID-19 pandemic	-Gaming disorder risk may increase during this pandemic because of the increased opportunity to play video -Parents -adolescent interaction is needed to regulate gaming time among adolescents	
G.	Clemens et al., 2020^5^	NS	Reflection on the potential effects of “social” distancing measures and school lockdown on child and adolescent mental health	Editorial	Negative effects of social distancing and school lockdown on mental health	Increased levels of mental stress which could erupt to interpersonal violence among adolescents	
H.	Fergert et al., 2020^6^	NS	Assessment of the key challenges and concerns for treatment and research on child and adolescent psychiatry (CAP) across Europe	Narrative review	Negative psychological effects of COVID-19 lockdown among adolescents	-Massive stress -Fear of death of relatives	
I.	Deslandes a nd Coutinho, 2020^21^	NS	To discuss the implications of social isolation for the intensive use of the internet among children and adolescents and its possible consequences for the practice of self-inflicted violence	Perspective	Negative effects of social isolation on self-harm and violence among adolescents during the COVID-19 lockdown	-Excessive internet usage could result to Behavioral Addictive Disorders (BADs) -Intensive internet usage could increase the vulnerability of adolescents to self- harm and violence	
J.	Imran et al., 2020^22^	NS	Assessment of the mental health challenges faced by children and adolescents during the COVID-19 pandemic and interventions required.	Review	Negative mental health effects of COVID-19 pandemic among adolescents	-Social distancing measures can result to domestic abuse -Increasing levels of depression, anxiety, and sleep deprivation.	
K.	Andrews et al., 2020^23^	NS	To determine the effect of peer influence on adolescent risk behaviors and its positive impact on young people to follow social distancing measures	Review	Adolescent peer group negatively influence the adoption of COVID-19 social distancing measures	-Fear of exclusion	- Peer influence could positively affect prosocial behavior regarding COVID-19.
L.	Golberstein et al., 2020^24^	NS	To assess the potential implications of school closure on adolescents' health, and mitigating factors	Opinion	Negative effects of COVID- 19 on adolescent health	-Worsening of existing mental health problems among adolescents -Disruption of school-based mental health services due to the lockdown.	-
M	Zhou et al., 2020^25^	China	To assess the prevalence of two specific mental symptoms, anxiety and depression, and their socio-demographic correlates among adolescents in the Chinese population	Full research article/ Cross- sectional	Mental health effects of COVID-19 among Chinese adolescents	-Depression and anxiety -Higher levels of anxiety and depression is commoner among late adolescents than other groups	
N.	Green, 2020^26^	NS	To assess the risks with which young people are faced during COVID-19 pandemic	Editorial	COVID-19-related risks of young people	-Increased vulnerability - Increased alcohol consumption which is likely to increase the frequency of domestic violence	
O.	Lindberg et al., 2020^27^	NS	To assess sexual and reproductive health of adolescents and young adults during the COVID-19 Pandemic	Opinion	COVID-19 influences the sexual and reproductive health of adolescents	-Disruption in romantic and sexual relationships - Reduced uptake of reproductive health services such as reproductive health education, spacing of childbirth, care for sexually transmitted infections, and maternal and child health for adolescents who were nursing children	-Reduced frequency of Intimate Partner Violence (IPV)
P.	Xiang et al., 2020^28^	NS	To explore the impact of COVID-19 pandemic on children and adolescents' lifestyle behavior	Letter-to-the- editor	Drastic negative lifestyle behaviors exist due to COVID-19	-Physical inactivity and sedentary time increased during the COVID-19 pandemic	
Q.	Wang et al., 2020^29^	NS	To assess the mitigation of the home confinement on children during the COVID-19 outbreak	Letter-to-the- editor	The negative effects of COVID-19 can be mitigated by the government		-Effectiveness of online learning -Mobilization of resources for healthy lifestyle and psychosocial support programs for schools
R.	Oosterhoff et al., 2020^30^	United States	To determine associations between social distancing motivations and mental and social health.	Full research article/ cross- sectional	Motivation of social distancing is associated with mental and social health	Reduced motivations to engage in social distancing and proactive measures for COVID-19 among adolescents, and associated with their mental and social health	
S.	Witt et al., 2020^31^	NS	To assess the challenges and opportunities of child and adolescent mental health service provision and research during the COVID-19 pandemic	Editorial	Challenges and opportunities in the provision of adolescent mental health service during the COVID-19 lockdown	-Reduction in mental health care	-Improved uptake of telepsychiatry

The negative effects of the COVID-19 lockdown were reported in 17 of the reviewed literature. These negative effects included an increased risk of trauma, anxiety, depression, and other mental illnesses. A high likelihood of interpersonal violence among adolescents was identified to erupt from increased levels of mental stress and increased alcohol consumption. An exercebation of mental health conditions was obtainable, alongside a reduction and/or disruption in mental health care during the COVID-19 lockdown. Also, reduced mental and social health of adolescents reduced their motivations to engage in social distancing and other proactive measures for COVID-19.

Gaming disorders among adolescents increased during the COVID-19 lockdown due to the increased opportunity for video games. Excessive screen time was associated with increased stress, poor sleep, increased risk of mental illnesses, and Attention Deficit Hyperactivity Syndrome (ADHS) among adolescents. Physical inactivity and increased levels of sedentary behavior increased the risk of fatty food intake which placed adolescents at risk for cardiometabolic complications and psychological disorders. A reduced uptake of reproductive health services such as reproductive health education, spacing of childbirth, care for sexually transmitted infections, and maternal and child health for adolescents who were nursing children was also observed during the COVID-19 lockdown period.

## Discussion

The positive effects of the COVID-19 lockdown identified in this study were outnumbered by the negative effects. This is not to suggest that lockdown measures should not be implemented, however the resulting negative effects may be worse and long-lasting than the pandemic itself. The positive effects of the COVID-19 lockdown were identified in 7 of the reviewed literatures. A decline in the intake of fast food was observed among adolescents during the COVID-19 lockdown period. A likely explanation for this occurrence is the increased time spent with family members in the home, thereby resulting to increased time set apart for making whole meals. Due to adolescents' confinement at home, a reduction in the intake of fast foods, and an increased intake of legumes, fruits, and vegetables was observed. This change in dietary pattern supports healthy growth among adolescents, and reduces the risk of development of cardiovascular disorders later in life. The consumption of such protective diets as legumes, fruits, and vegetables which we identified is contrary to reported diets during the Severe Acute Respiratory Syndrome and Ebola virus confinement periods^32^,^33^.

We also identified increased physical activity and reduced sedentary behavior among persons in mid-childhood to mid-adolescent periods during the COVID-19 lockdown. This finding could be due to the lack of totally engaging activities during COVID-19 lockdown as compared to the pre-lockdown period. It could also be due to the enhanced effectiveness of online schooling which primarily targets improved mental and physical activities for in-school adolescents^29^. Online schooling has been reported to yield optimal academic performance among adolescents aged 10–15 years compared to those aged 16–19 years. However, online schooling was only suitable for in-school adolescents, with greater peculiarity to adolescents who had examinations in view prior to the commencement of the lockdown period. Due to the cost attached to the registration of adolescents on online schooling modalities, adolescents from low middle-income countries could not have benefitted optimally from online school compared to their counterparts in high-income countries. Although this finding was not captured in the present review, the existing socioeconomic inequalities across these locations presents anecdotal evidence in this regard. In spite of the differences observed in the effects of online schooling across the different adolescent age groups, exercises during the COVID-19 lockdown yielded huge benefits across board. Both government and private schools have gained psychosocial support required for the maintenance of a healthy lifestyle for their students who are mostly adolescents^29^. In other instances, older adolescents intentionally engaged in group exercises to ensure fitness and healthy living. Thus, increased physical activity and psychosocial support for adolescents through their schools helped to maintain their body glucose level within the normal range after two weeks of implementation of the lockdown.

This study found an improved uptake in telepsychiatry during COVID-19 lockdown. The lockdown period limited access to clinics for consultations. However, the period provided an opportunity for telepsychiatry, a health care strategy which had not been maximally utilized previously. Due to the telepsychiatry avenue, adolescents with mental challenges could reach out to mental health experts both for first-time and follow-up consultations^31^.

Findings from this study revealed that varying degrees of mental health challenges such as anxiety and depression could have resulted from the lockdown. The lockdown : was associated with an increased risk of trauma and domestic abuse. Qualitative studies have similarly identified a range of psychological effects of confinement during the SARS and Ebola outbreaks, including frustration, boredom, grief, and anxiety-induced insomnia^32^,^33^. Also, quarantine measures during the Severe Acute Respiratory Syndrome outbreak similarly had psychological health challenges such as post-traumatic stress disorder and depression^34^ In a survey conducted among Chinese adolescents aged 12–18 years, a high prevalence of depression, anxiety, and combined depression and anxiety was reported during the COVID-19 lockdown^35^. Findings from a review similarly reported depression, anxiety, and stress among adolescents.^18^ The results of a survey conducted among adolescents aged 12–18 years in India reported a high prevalence of anxiety (31%), depression (43%), and depression-anxiety comorbidity (31%)^25^. A high prevalence of anxiety and depression was similarly reported by Cao et al. in their study.^36^

Previous studies corroborate our findings on the existence of fear and confusion among adolescents especially pertaining to sudden separation and school closure^37^. The similarities in these findings indicate that lockdown or confinement measures during health event of concern could pose significant risks to individual's mental and psychological well-being. The manifestations of psychological impacts of the lockdown may be hidden among adolescents, hence the need to propose measures which enhance their mental health.

We found that the COVID-19 lockdown could have negatively influenced dietary habits. We noted an increased average intake of fried and sweet foods, the consumption of which causes accumulation of cholesterol, and result to excessive body gain or obesity during this period. A recent study conducted to assess energy intake among Undergraduate Australian students during the COVID-19 pandemic reported an increase in snacking frequency, and energy density of consumed snacks^37^. The paucity of findings on dietary habits in low middle-income countries such as Nigeria limits the generalizability of the effects of the COVID-19 lockdown on adolescents' nutrition. Our findings thus indicate that an increase consumption of unhealthy calorie-dense foods occured among adolescents during the lockdown. Also, the home environment affords the opportunity for consumption of healthy and nutritious diets which are essential for growth and development of adolescents.

We also noted increased physical inactivity which predisposes adolescents to sedentary behavior during the COVID-19 lockdown. This exposed adolescents to increased screen time which could worsen mental health issues, and result to sleep deprivation. Due to the absence of parental monitoring, adolescents became more prone to watching violent videos and accessing websites that could result to the infliction of self-harm. Although online schooling recently gained recognition in the continuation of formal education during the lockdown, excessive internet usage could produce behavioral addictive disorders. Increased levels of addictive disorders has been identified as one of the consequences of disasters among adolescents^38^. This identifies the need for controlled internet usage under parental supervision.

In a bid to coping with the lockdown, we found that adolescents were exposed to the risk of gaming disorders. Gaming disorders arise from prolonged hours spent on games. The likelihood of internet addiction characterized by poor controlled access to the internet or computer usage that result to mental impairment is enhanced during lockdowns^38^,^39^. There exists an association between online games and internet addiction, and time spent on these games correlate with the level of depression, anxiety, and sleep deprivation^38^,^39^. It could also result to ADHD^40^. This indicates that adult guidance is required both in the selection of games, and the allocation of time for such games.

In this study, we noted the likelihood of emotional implications of the COVID-19 lockdown among adolescents. Our findings revealed a disruption in romantic and sexual relationships among adolescent. Although this offered some form of protection for adolescents, there also existed the risk of loneliness which could negatively affect diet and sleep patterns, and eventually result to mental disorders. In many instances, assault, increased levels of aggression, sexual violence, and rape were also observed in the studies examined in this review. Similar effects of lockdown on emotional relationships have been reported^39^. This implies that lockdowns could impair the emotional health of adolescent, and this could negatively affect their overall health and wellbeing. For instance, events of rape resulted in traumatization and post-traumatic stress disorder which could impair adolescents' sense of identity. Also, physical violence could have caused fear, depression, and other levels of psychological or emotional illnesses especially among younger adolescents. Evidence from this review therefore posit that the COVID-19 lockdown period could have had significant effects on emotional and psychological health and well-being of adolescents, and could persist further into adulthood.

## Strengths and Limitations

The findings in this study could have been limited by the paucity of available literature on the effects of the COVID-19 lockdown on adolescents from low middle-income countries at the time the review was done. In addition, the unavailability of literature on the experience of out-of-school adolescents on exercise and online schooling could have limited the generalizability of the findings in this review. Overall, this review presented credible results regarding the global impact of the COVID-19 lockdown on adolescents' health.

## Conclusion

Although the lockdown measure was adopted to reduce further transmission of COVID-19, it similarly yielded negative results among different population groups. Adolescents are vulnerable groups who are equally subjected to the consequences of the COVID-19 lockdown. There exists an intricate relationship between all aspects of their health, with one directly influencing the other. The risks to which adolescents were exposed especially during the COVID-19 lockdown impacts their health during adulthood. Therefore, we recommend parental supervision of adolescents' screen time. Also, awareness campaigns on health education programs health should be continually communicated to adolescents via the available traditional and modern media. In addition, adolescent health should be prioritized by policymakers to ensure that future lockdowns do not have adverse effects on their health. To adolescents who serve as heads of households or breadwinners, social protection should be ensured by policy makers to enable the protection of their right to good health and wellbeing. Furthermore, the integration of adolescent-focused mental health services via teleconsultation should be adopted in the effective management of adolescents' mental health. Moreover, families should be responsible in the provision of adequate essential nutrients for adolescents in their diets, while promoting home-based physical exercise.
